# HAND GRIP STRENGTH ASSESSMENT IN A HOME-DELIVERED MEAL PROGRAM: FEASIBILITY AND PRELIMINARY FINDINGS

**DOI:** 10.1093/geroni/igz038.3405

**Published:** 2019-11-08

**Authors:** Heather L Hutchins-Wiese, and Sarah E Walsh

**Affiliations:** Eastern Michigan University, Ypsilanti, Michigan, United States

## Abstract

Declining hand grip strength is associated with adverse health outcomes and is a relatively quick and easy-to-administer functional assessment; however, grip strength is not routinely assessed in home-delivered meal (HDM) programs. The purpose of this sub-study was to test if grip strength assessment is feasible and useful in a HDM population. Among HDM clients (n=258) who completed health assessments between 2016 and 2018, a voluntary convenience sample of 34 HDM clients (23 women, 11 men) participated in the grip strength assessment sub-study. Sub-study participants were younger (72.2+/-7.35 vs. 77.0+/-10.50 years), with no other significant differences compared to the full sample of HDM participants. The average maximum grip strength was 21.99+/-6.97 kg for the dominant hand; 16 clients were categorized as having normal, 6 intermediate, and 12 weak grip strength. Normative categories for grip strength vary by gender because men typically have higher grip strength measures than women. In this population, more men were categorized as having weak grip strength compared to women (X (2, 34) =11.44, p=.03). In men, hand grip strength tended to be lower in those who reported a fall in the previous 6 months compared to those who did not report a fall (20.8+/-7.01 vs 29.3+/-6.32, p=.079). The gender differences and 8 kg difference in grip strength relative to reported falls are clinically meaningful in this vulnerable population. Future research is needed with a larger sample of HDM clients to confirm these preliminary findings.

